# Spatiality, seasonality and ecological risks of heavy metals in the vicinity of a degenerate municipal central dumpsite in Enugu, Nigeria

**DOI:** 10.1186/s40201-015-0168-0

**Published:** 2015-03-10

**Authors:** Kanayochukwu C Ajah, Joel Ademiluyi, Chidozie C Nnaji

**Affiliations:** Department of Civil Engineering, University of Nigeria, Nsukka, Enugu State Nigeria

**Keywords:** Heavy metals, Solid waste, Health, Rainfall, Pollution

## Abstract

**Background:**

Improper waste disposal is responsible for the contamination of both surface and ground water resources. Heavy metals leached from improperly disposed solid waste constitute grave environmental and health hazards because of their toxic and persistent nature. There are thousands of open dumps in Nigeria one of which is the Enugu State Waste Management Authority dumpsite.

**Method:**

Forty sampling nodes were systematically established around the Enugu State waste Management Authority central dumpsite located at Ugwuaji, Enugu State, Nigeria. Ten heavy metals (arsenic, cadmium, cobalt, copper, chromium, iron, lead, manganese, nickel and zinc) were sampled at different depths of each node in both rainy and dry seasons.

**Result:**

Iron and lead were the predominant metals in the vicinity of the waste dump with average values of 132.10 mg/kg and 117.52 mg/kg respectively. The order of abundance of the ten heavy metals monitored is Pb > Fe > As > Zn > Cu > Co > Ni > Cd > Cr > Mn. Generally, there was significant correlation (0.25 to 0.74) among all the metals except between cobalt and manganese in the rainy season. In the dry season, all the metals were significantly correlated (0.29 to 0.813) except for copper and lead, copper and arsenic, zinc and arsenic, and cobalt and manganese. The concentrations of most of the heavy metals approached a constant level at a depth of 1 m. On the other hand, the concentrations of arsenic, cobalt and iron continued to decrease even at a depth of 2 m. The pollution loading index values for the soil are 1.706 for rainy season and 2.54 for dry season.

**Conclusion:**

The high pollution loading index represents a significant level of deterioration. It can be concluded that the dumpsite constitute a serious environmental and health hazard.

## Introduction

One of the most menacing challenges facing developing countries is an ever ballooning quantity of waste generation without commensurate facilities and resources to face this challenge. Inability of waste management authorities to cope with waste generated and consequent indiscriminate disposal of waste has turned many erstwhile beautiful Nigerian cities into mega ghettos. The result is unmitigated pollution of land, air and water which exposes the populace to miasma of health hazards. There is no doubt that a healthy environment has a high correlation with human health. Air pollution usually results from industrial and domestic emissions, water contamination results from industrial effluent discharges, agricultural runoff and sewage disposal; while soil pollution results from uncontrolled solid waste disposal on land. The two major concerns regarding waste disposal on land are: (i) surface and ground water contamination by leachate and (ii) bioaccumulation of toxic heavy metals in soil, uptake of these heavy metals by plants and biomagnifications of these metals up the food chain. Besides, heavy metals accumulation in soil can hamper soil productivity by interfering with soil fauna and flora. Most heavy metals naturally occurring in the earth crust as trace elements are usually found buried deep in the heart of the earth. However, massive exploitation of natural resources has given rise to a build-up of these toxic elements in the human environment.

Anthropogenic sources of heavy metals include: emissions from vehicle exhaust pipe, tyre wear particles, weathered street surfaces, brake lining wear particles, power plant combustion, metallurgical industry, auto repair shops, chemical plants, weathering of buildings and pavement surfaces, atmospheric deposits, mining, smelting, waste disposal, urban effluents, pesticides, fertilizers, sawdust disposal, herbicides, pharmaceuticals, batteries, fungicides, paints, pigments and dyes, leather tanning, photographic films, fireworks, printer and photocopier toners, cement, candles, rubber, etc. These are commonly used products with which humans come in contact on a daily basis, and which constitute a substantial part of waste at dump sites. Several researchers have found elevated levels of heavy metals in street dusts [1-5] agricultural soils [6-8], cemetery [9] solid waste dumps [10], oil and gas facilities [11] and lake sediments [12]. Chemical and physical affinity of metal ions for various waste materials may reduce their leachability, however, metal ions mobility increases over time as acidic and oxidizing conditions prevail [13]. It has been suggested that soil acidity be used as basis for evaluating soil contamination by several elements [6]. Potential binding ligands include carbonates, chloride, dissolved organic matter, colloidal solids and sulfide.

Heavy metal toxicity is determined by route, pattern and duration of exposure. Routes of exposure to heavy metals include (i) ingestion of soils, contaminated water, vegetables and fruits grown on contaminated soils, and animals that grazed on contaminated areas; (ii) inhalation of soil particles, dust and fumes and (iii) dermal contact [14-16]. Drinking of contaminated water and consumption of agricultural products represent an important source of heavy metals ingestion. Accumulation of heavy metals in agricultural products results from unwholesome practices such as use of domestic and industrial effluent for irrigation, cultivation of plants on waste dumps and surrounding soils, and grazing of animals on grasses growing on contaminated soils. It is common practice in Nigeria and some other developing countries for people to grow their crops on waste dumps and on soils where raw sewage is discharged [17,18]. Currently, there is no official policy to stop these practices or sensitize the masses on the dangerous implications of these practices. Leachability and uptake of heavy metals by plants are soil and plant specific. While leafy vegetables exhibit preferential uptake of cadmium and copper, cigarette leaves can accumulate large amounts of arsenic and cadmium, arsenic and lead. Elevated levels of arsenic (0.5 – 7.5 mg/kg) have been found in rice and vegetables grown in Chenzhou City of Southern China [19]. Several health hazards have been associated with consumption of high doses of heavy metals [20,21]. These health hazards range from mild illnesses such as ulcers, diarrhea, nausea, abdominal pain, gastrointestinal disorders, respiratory disorders, cough, nervous disorder, psychological disturbances to life threatening diseases such as cancers, cardiovascular diseases, asthma, kidney and liver damage, coma and diabetes.

In the light of the aforementioned realities, there is need for a detailed study of heavy metal contamination of the soils in the vicinity of the Enugu municipal dumpsite with a view to ascertain the extent of soil contamination.

## Methodology

### Description of study area

Enugu state is one of the five Southeastern states of Nigeria, located between latitude 6°.00’N and 7°.00’N and longitude 7°.00’E and 7°.45’E. It falls within the humid tropical rainforest belt of the Southeastern Nigeria. It has two distinct seasons: dry and rainy seasons. The rainy season commences in April and ends in October, followed by the dry season. The annual rainfall ranges between 937.2 mm to 2243.3 mm while the temperature ranges between 20.3°C to 32.16°C [22,23]. The 2006 census put the population of Enugu at 722, 664 [22]. Enugu State Waste Management Authority (ESWAMA) municipal solid waste (MSW) disposal site of approximately 7.878 ha of land space, is located in the southern part of Enugu Metropolis with its geographic position system (GPS) coordinates as: Elevation: 186 m; North: 6°26.27’; and East: 7°32.831’.

**Figure 1:**
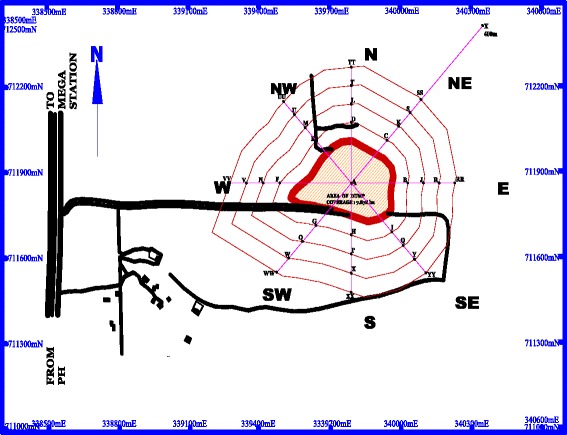
**Map showing Enugu state waste management authority waste dump and environs.**

The dump site is about 1.6 kilometers away off Enugu-Port Harcourt expressway as shown in Figure 1. The site slopes gently downwards away from its centre in all directions into the environs. The dumpsite is the final disposal ground for all wastes (domestic, construction/demolition, industrial and agricultural) generated in Enugu metropolis. The dumpsite was originally conceived as a landfill but has degenerated to a massive open dump because of poor management, inadequate manpower and lack of requisite technology. The bottom is not lined for leachate containment, and no compaction is undertaken. There is no perimeter fencing, hence scavengers and stray animals roam the dumpsite unrestricted. ESWAMA is supposed to be a waste management authority but what it essentially does is to undertake waste collection within Enugu metropolis and disposal of collected waste at the central dumpsite. The waste does not undergo any level of treatment or processing before disposal. There is no formal sorting for material recovery and recycling. Informal material recovery is undertaken by scavengers who sometimes set newly deposited heaps of waste ablaze in a bid to recover valuable metal waste. According to [24,25], the dump site lies on the massive, dark coloured Enugu shale formation predominantly made up of shales, clays, silts and limestones. It is overlain by the Mamu formation and underlain by the Awgu shale formation. This shale formation is made up of porous clays and impermeable shale units. Nevertheless, the weathered top soils are permeable, but are not of great thicknesses; hence they cannot yield appreciable quantities of water to boreholes. As a result of the afore-mentioned hydrogeologic characteristics of Enugu Metropolis and environ, shallow dug wells are the only sources of groundwater supply.

### Sample collection

For the purposes of data collection, the area was divided into eight equal segments of 45° each. Concentric loops were then introduced at equal distances of 20 m from each other starting from the boundary of the dump site. In order to determine the number of loops required before sampling could commence in earnest, trial soil samples were collected at nodes (formed by the intersection of the concentric loops and 45° radial lines) equally spaced at 20 m intervals from the boundary of the dumpsite, along the radial line AE. When two nodes on two successive loops showed no significant difference in pollutant concentration, additional loops were no longer necessary. Hence, concentric loops equally spaced at 20 m beginning from the boundary of the dumpsite were established and detailed sampling commenced. Soil samples were collected at the centre (A) of the dumpsite and at the nodes formed by the intersection of the loops with the 45° radial lines. Soil samples were collected at the nodes by means of auger bits at depths of 0.5 m, 1 m, 1.5 m and 2 m. This implies that for each node, samples were collected at four sampling depths. Hence there are 164 sampling points for contaminated soil. In order to obtain the background levels of heavy metals in the soil, soil samples were obtained from point X located 400 m away from the boundary of the dump site (see Figure 1). Soil samples collected were bagged in transparent polythene bags and then sent to the laboratory for heavy metals analyses. The samples were oven dried and then ground to a fine texture using pestle and mortar in the laboratory. The finely ground soil samples were sieved and 1 g was used for digestion. Supra pure-merck nitric acid and hydrogen peroxide (30%) were used for the digestion in an open vessel. Soil sample were analysed for each heavy metal using a calibrated atomic absorption spectrophotometer (AA320N). For the purpose of this research, two periods were selected, viz: dry season (October – March) and wet season (April – September). During these two periods, soil samples were collected and analysed in the laboratory. This approach produced results that are more representative of the actual field situation in the dump site. The metals monitored are iron, copper, lead, zinc, arsenic, cobalt, nickel, chromium, cadmium and manganese.

Undisturbed soil samples were also collected at depths of 0 to 1 m and 1 – 2.5 m in four (4) randomly selected locations for physical characterization. Based on the American Association of State Highway and Transport Association of State Highway and Transport Officials (AASHTO) classification of soil, the soil type of the study area is reddish, sandy and silty clay A-2-6 which is locally called lateritic soil. The soil has the following parameters: percentage passing No 200 sieve (26.8%), liquid limit (26.2), plastic limit (9.5), plasticity index (16.7), moisture content (12.5%), bulk density (2.21 g/cm^3^), dry density, (1.98 g/cm^3^), specific gravity (2.41) and porosity (0.36). Result of soil characterization by [26] within this vicinity showed that the soil contains 55% gravel, 13% sand, 18% silt, 14% clay.

### Data analyses

Laboratory results were further subjected to statistical analyses, in order to facilitate interpretation. Using Microsoft Excel, the data was subjected to descriptive statistical analyses. Correlations between pairs of metals were also obtained. Geostatistical methods were employed to obtain the spatial variation of the various heavy metals in the dumpsite and environs.

Since the metals were sampled at only finite and discrete number of points, it was necessary to estimate metal concentration at other points where samples were not taken. The semivariogram (γ_(k)_) function was adopted. It is defined as follows [27,28]:

1 $$ {\gamma}_{(k)}=\frac{1}{2n(k)}{\displaystyle \sum_{i=1}^{n(k)}{\left[Z\left({x}_i\right)-Z\left({X}_{i+k}\right)\right]}^2} $$

Where γ_(k)_ is the square of the difference between a soil property (metals in this case) at a point *x*_*i*_ and the same soil property at another point located at a distance *x*_i+k_, and *n(k)* is the number of pairs of observations separated by a lag distance of k. Three-dimensional contour maps of heavy metals distribution were drawn by the Krigin method of point interpolation using Surfer 11.

Risk assessments were performed using already established indices such as the pollution index, geoaccumulation index and ecological risk index. Pollution index was calculated as the ratio of the mean concentration of each heavy metal to the baseline or background concentration. It is defined as follows [7]:

2 $$ PI=\frac{C_i}{S_i} $$

Where *C*_*i*_ is the average concentration of individual metal in the dumpsite and *S*_*i*_ is the baseline concentration. PI values < 1 indicate low level of pollution, 1 ≤ PI ≤ 2 indicate moderate level of pollution, 2 ≤ PI ≤ 5 indicate high level of pollution, while PI ≥ 5 indicate extreme pollution level.

Another index, the geoaccumulation pollution index was also used to assess the degree of soil contamination by metals. The geoaccumulation index (*I*_*geo*_) is defined as [7,29]:

3 $$ {I}_{geo}=Lo{g}_2\left(\frac{C_i}{1.5{S}_i}\right) $$

*I*_*geo*_ < 0 indicates pristine or uncontaminated state, 0 ≤ *I*_*geo*_ ≤ 1 indicates uncontaminated to moderately contaminated state, 1 ≤ *I*_*geo*_ ≤ 2 indicates moderately contaminated state, 2 ≤ *I*_*geo*_ ≤ 3 indicates moderately to heavily contaminated state, 3 ≤ *I*_*geo*_ ≤ 4 indicates heavily contaminated state, 4 ≤ *I*_*geo*_ ≤ 5 indicates heavily to extremely contaminated state, and *I*_*geo*_ > 5 indicates extremely contaminated state.

Since PI values give the pollution status of the site with respect to individual metal, overall pollution status of the site was determined using the pollution loading index (PLI) calculated as follows, for *n* number of metals [29].

4 $$ PLI={\left({\displaystyle \prod_{i=1}^nP{I}_i}\right)}^{\frac{1}{n}} $$

*PI* has been defined by Equation 2.

Ecological risk index (*ERI*) was used to assess the level of risk posed by the heavy metals using the heavy metal toxic factors of [30]. *ERI* is defined as follows [19]:

5 $$ ERI={\displaystyle \sum_{i=1}^n{T}_i\frac{C_i}{B_i}} $$

Where *C*_*i*_ has been previously defined, *T*_*i*_ is heavy metal toxic factor for a given metal, *B*_*i*_ is the guideline value for the metal and *n* is the number of metals. Toxic response factors used are: Cd(30), Cu(5), Cr(2), Zn(1), AS(10), Co(5) and Ni(5) in mg/Kg [31]. Heavy metals guideline values of the Department of Petroleum Resources were used, and they are as follows: Cu(56), Zn(140), Pb(85), Cd(0.8), Ni (35), Cr (100), Co (20) and As (29) in mg/Kg [32]. *ERI* are classified as follows: low contamination (*ERI* ≤ 50), moderate contamination (50 ≤ *ERI* ≤ 100), considerable contamination (100 ≤ *ERI* ≤ 200) and high contamination (*ERI* > 200). Finally, an attempt was made at source identification by hierarchical cluster analysis using the Statistical Package for Social Sciences (SPSS 16.0).

## Results and discussion

### Heavy metals concentration in dumpsite soil

Results obtained from field studies indicate very clearly the preponderance of heavy metals in dumpsite soil. Table 1 shows that iron and lead were the predominant metals in the vicinity of the waste dump with average values of 132.10 mg/kg and 117.52 mg/kg respectively. The order of abundance of the ten heavy metals monitored is Pb > Fe > As > Zn > Cu > Co > Ni > Cd > Cr > Mn. Many previous researchers have found that the concentration of iron and lead in soil were higher than the concentration of other metals. The order of abundance of these heavy metals displayed slight seasonal variation as shown below.

**Table 1:** **Seasonal descriptive statistics of heavy metals**

	**Rainy season**							**Dry season**						
**Heavy metal**	**Mean**	**S.D.**	**Variance**	**Kurtosis**	**Skewness**	**Min**	**Max**	**Mean**	**S.D.**	**Variance**	**Kurtosis**	**Skewness**	**Min**	**Max**
Copper	25.17	8.92	79.60	-0.76	0.18	7.50	45.00	87.77	72.96	5322.71	-0.03	0.81	7.50	311.00
Zinc	43.37	11.71	137.21	1.09	0.47	12.50	85.00	76.37	62.56	3914.28s	8.47	2.55	12.50	431.75
Lead	138.48	57.99	3362.76	0.18	0.74	42.50	357.50	125.72	61.10	3732.78	-0.21	0.48	18.00	324.25
Arsenic	96.44	62.46	3901.73	-0.54	0.43	0.00	247.50	90.63	60.99	3720.22	-0.72	0.39	0.00	245.00
Chromium	11.40	10.47	109.57	17.90	3.58	2.50	80.00	18.34	21.76	473.54	16.55	3.48	2.50	162.50
Nickel	34.19	41.37	1711.12	11.36	3.13	0.00	242.50	25.49	21.11	445.83	0.81	1.17	0.00	92.00
Cadmium	20.15	24.92	621.26	17.72	3.45	0.00	192.50	21.55	22.87	523.14	23.19	3.63	0.00	192.50
Iron	113.79	132.24	17486.78	8.85	2.76	2.50	706.00	121.26	104.39	10896.94	6.22	1.88	2.50	677.50
Cobolt	51.65	63.54	4087.48	15.00	3.18	2.50	475.00	45.23	46.45	2157.72	3.42	1.90	2.00	232.50
Manganese	6.44	14.12	199.34	21.88	4.45	0.00	97.50	12.28	12.81	163.97	7.91	2.18	0.00	85.00

Pb > Fe > As > Co > Zn > Ni > Cu > Cd > Cr > Mn (rainy season).

Pb > Fe > As > Cu > Zn > Co > Ni > Cd > Cr > Mn (dry season).

A summary of the order of abundance of anthropogenic heavy metals in the soil as obtained by various researchers has been presented in Table 2.

**Table 2:** **Order of heavy metal anthropogenic-induced abundance in the soil**

**Source**	**Location**	**Order of abundance**	**Reference**
Agricultural soil	Chelopech, Bulgaria	Fe > Cu > Zn > Pb > Cr > As > Ni > Co > Cd > Hg	[6]
Agricultural soil	China	Zn > Cr > Pb > Cu > Ni > As > Cd > Hg	[7]
Agricultural soil	Xiamen, China	Mn > Zn > Pb > Cr > As > Cu > Ni > Cd > Hg	[8]
Alluvial soil	Japan	Zn > Cu > Pb > Ni > Cd	[33]
Cemetery	Kigali, Rwanda	Cr > Zn > Pb > As > Cu > Al > Fe	[9]
Dune soil	Japan	Zn > Cu > Pb > Ni > Cd	[33]
Flood plain	Biesboch, Netherlands	Zn > Pb > Cu > Cd	[34]
Gas plant	Niger Delta, Nigeria	Fe > Mn > Zn > V > Cr > Cu > Pb > Ni > Cd > Hg > As	[11]
Industrial areas	Gujarat, India	Ba > V > Cr > Sr > Cu > Zn > Ni > Co	[35]
Irrigated soil	Bahr El-Baker, Egypt	Cd > Cu > Zn > Cr > Ni > Pb	[36]
Jute mill waste dump	Assam, India	Fe > Zn > Pb > Cu > As > Cd > Cr > Ni	[37]
Lake sediment	Dongting, China	Zn > Cr > Cu > Pb > As > Cd > Hg	[12]
Metal mining and smelting site	Baiyin, China	Cr > Zn > Pb > Cu > As > Cd	[38]
Metal scrap dump	Delta State, Nigeria	Fe > Zn > Cr > Cu > Pb > Co > Ni > Cd	[39]
Oilfield	Niger Delta, Nigeria	Fe > Mn > Zn > V > Cr > Pb > Cu > Ni > Cd > Hg > As	[11]
Pipeline	Niger Delta, Nigeria	Fe > Ni > Mn > Zn > V > Cr > Pb > Cu > Cd > Hg > As	[11]
Red soil	Japan	Zn > Ni > Cu > Pb > Cd	[33]
Roadside	Botwana	Fe > Al > Ni > Mn > Pb > Zn > Cu > Co	[1]
Roadside	Urumqi, China	Cd > Zn > Cu > Pb > Be > Ni > Mn > Cr > Co	[2]
Roadside dust	Ilorin, Nigeria	Fe > Mn > Pb > Cr > Zn > Cu	[3]
Solid waste dump	Ibadan, Nigeria	Pb > Cr > Ni > Co > Cd	[10]
Street dust	Tehran, Iran	Fe > Mn > Zn > Pb > Cu > Ni > Cr > Cd > Li	[5]
Street dust	Cairo, Egypt	Zn > Pb > V > Cr > Ni > Co > Ag > As > Cd	[4]
Top soil	Fuyang, China	Zn > Cu > Pb > Ni > Cd	[40]
Urban road dust	China	Zn > Pb > Cu > Cr > Ni > Cd	[7]
Urban soil	China	Pb > Zn > Cu > Ni > Cr > Cd	[7]
Urban soil	China	Zn > Pb > Cu > Cd	[41]
Urban soil	Poznań, Polland	Zn > Pb > Cu > Cd	[28]
Volcanic soil	Japan	Zn > Cu > Pb > Ni > Cd	[33]
Solid waste dump	Enugu, Nigeria	Pb > Fe > As > Zn > Cu > Co > Ni > Cd > Cr > Mn	This study

High level of iron and lead were found in agricultural soils in Bulgaria, alluvial and dune soils in Japan, flood plains in the Netherlands, around gas plants in Niger Delta, Nigeria, irrigated soils in Egypt, waste dumps in India and Nigeria, roadside dust in china, Botswana, Nigeria and Iran [1-6,10,11,33,34,36,37]. Lead accumulation can be attributed to low mobility and strong association to soil constituents such as organic matter, minerals of clay fraction, and oxides of iron and manganese [36].

To buttress this point, Table 3 below shows significant correlations of 0.68 between lead and iron, and 0.43 between lead and manganese. Lead (Pb) exists in many forms in the natural sources [42]. Its concentration in the human environment increased with the introduction of leaded gasoline in the 1920s. Lead accumulation in the surrounding soil is facilitated by the presence of large proportion of organic matter in disposed waste. Generally, there was significant correlation among all the metals except between cobalt and manganese in the rainy season. In the dry season, all the metals were significantly correlated except for copper and lead, copper and arsenic, zinc and arsenic, and cobalt and manganese. The most significant correlation was 0.813 between copper and zinc (rainy season), 0.74 between nickel and iron, and 0.7 between chromium and iron. Significant correlation between the metals might be attributed to commonality of source and their common tendency to persist in the environment.

**Table 3:** **Seasonal correlation of heavy metals**

	**Rainy season**										**Dry season**									
	***Cu***	***Zn***	***Pb***	***As***	***Cr***	***Ni***	***Cd***	***Fe***	***Co***	***Mn***	***Cu***	***Zn***	***Pb***	***As***	***Cr***	***Ni***	***Cd***	***Fe***	***Co***	***Mn***
*Cu*	1										1									
*Zn*	0.606	1									0.813	1								
*Pb*	0.542	0.59	1								0.189	0.37	1							
*As*	0.376	0.47	0.66	1							0.070	0.198	0.65	1						
*Cr*	0.372	0.56	0.61	0.68	1						0.457	0.63	0.52	0.43	1					
*Ni*	0.286	0.54	0.57	0.56	0.47	1					0.237	0.43	0.63	0.53	0.53	1				
*Cd*	0.299	0.54	0.59	0.61	0.66	0.61	1				0.291	0.4	0.52	0.52	0.5	0.42	1			
*Fe*	0.448	0.68	0.55	0.54	0.7	0.74	0.52	1			0.321	0.43	0.47	0.44	0.68	0.44	0.39	1		
Co	0.470	0.45	0.53	0.55	0.63	0.37	0.46	0.5	1		0.060	0.29	0.66	0.58	0.53	0.51	0.58	0.62	1	
Mn	0.250	0.37	0.43	0.33	0.26	0.32	0.54	0.31	0.055	1	0.602	0.37	0.37	0.33	0.43	0.43	0.57	0.3	0.145	1

**Figure 2:**
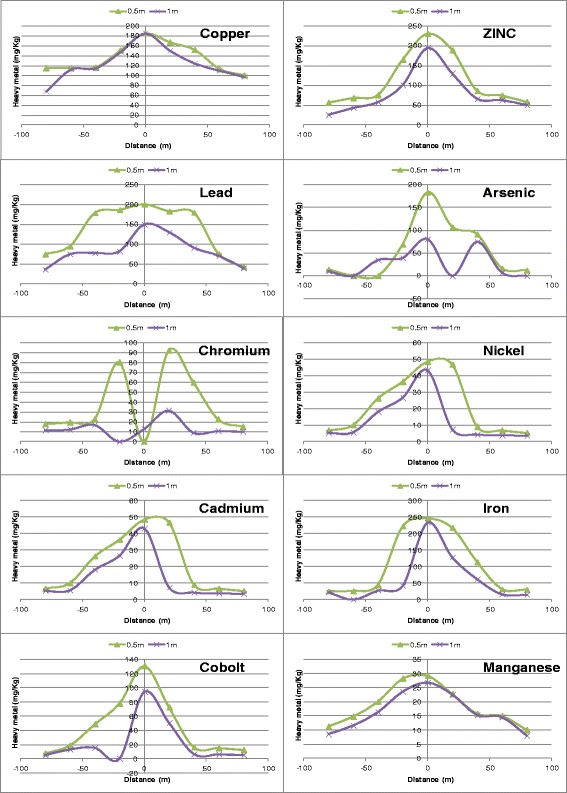
**Spatial distribution of heavy metals in dry season.**

**Figure 3:**
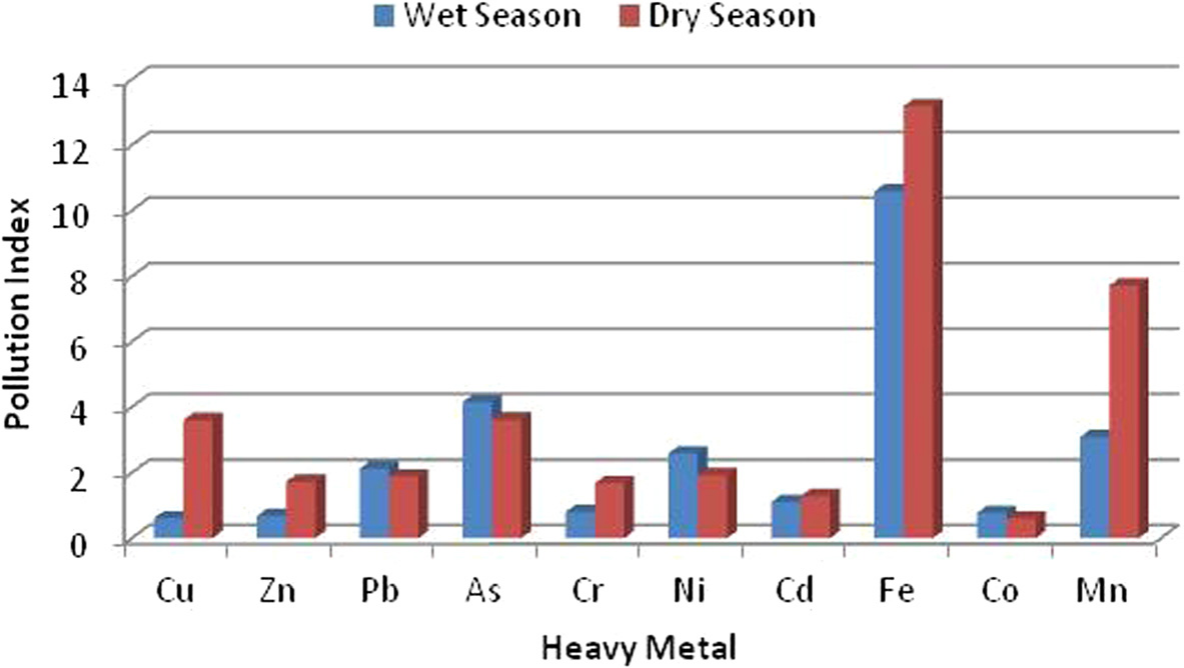
**Seasonal variation of pollution index.**

**Figure 4:**
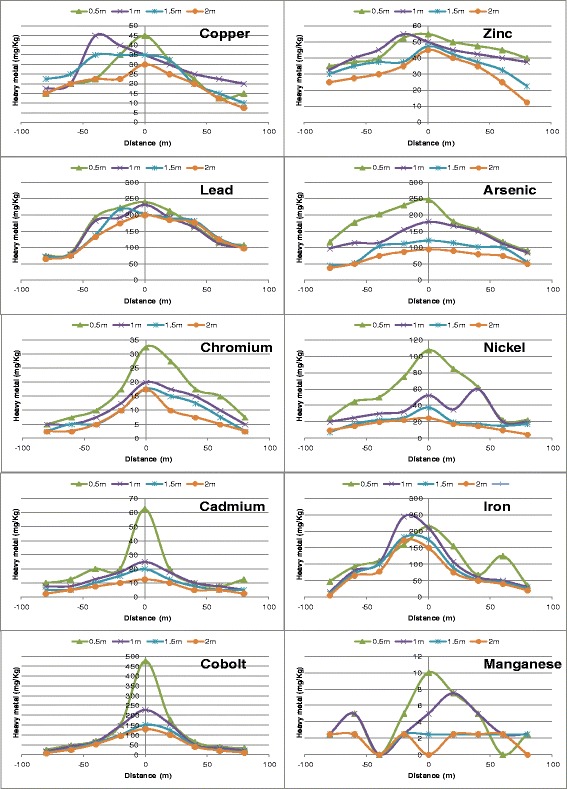
**Spatial distribution of heavy metals in rainy season.**

In almost all cases, concentration of heavy metals peaked at the dump site but gradually tailed to the environs. Figures 2 and 3 show that there was a gradual attenuation of lead in both lateral and vertical directions. This is evidenced by the undulating nature of the curves and by the closeness of the concentration lines for different depths. This can be attributed to the effect of leaching by infiltrating water. Besides, during heavy rainfall, runoff from the dumpsite spreads to the surrounding soil, thereby redistributing the heavy metals. The same phenomenon applies to copper and zinc as depicted by the curves. For most of the metals especially lead, cobalt, cadmium and iron, the vertical variation of metal concentration becomes almost insignificant at a distance of about 80 m from the dumpsite. This suggests persistence, and that the preferred direction of transport is downward. Moreover, the spatial variation of heavy metals in a given catchment area decreases with depth. This is easily visualized from Figures 2 and 4. Curves of heavy metals variation at 0.5 m depth are much more pronounced than those of 2 m depth. It has been observed that it is this persistence and low mobility of heavy metals in the environment, even under high precipitation, that aggravates the risk posed by heavy metals [43].

**Figure 5:**
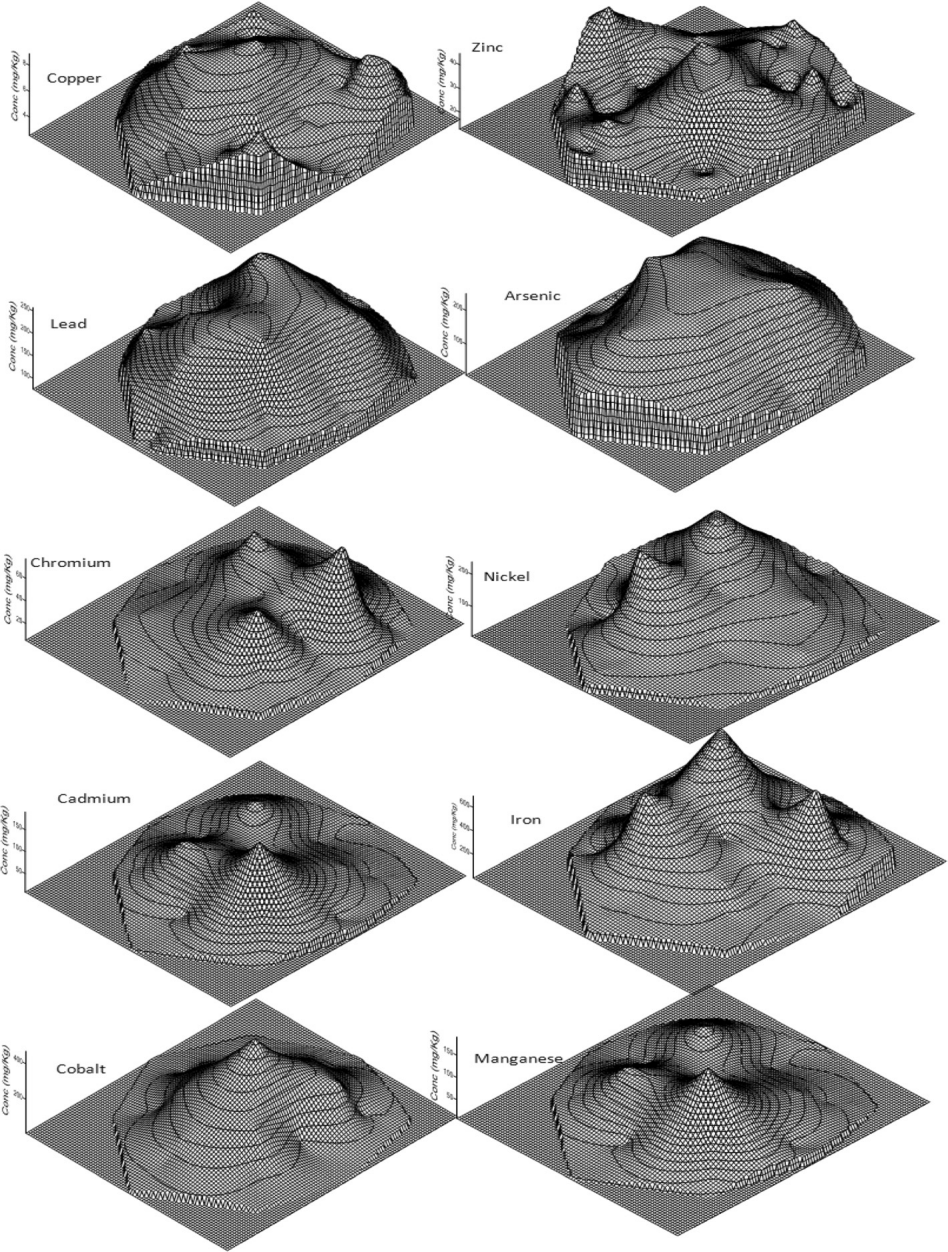
**Three dimensional maps of heavy metal distribution in study area.**

From Figures 2, 3 and 5, it can easily be deduced that cobalt, cadmium, nickel and chromium are the most persistent of all the heavy metals studied. Their very high concentrations within the dumpsite as well as the comparatively low concentration at lower soil depths suggest a tendency to persistence and accumulation within the dumpsite. The problem of heavy metal accumulation in dumpsites is a matter of serious concern particularly in developing countries where farmers prefer to cultivate vegetables in waste dumps because of their perceived fertility. Vegetables grown on such soils are usually very leafy and attractive to consumers. Paw paw fruits and maize cultivated around the dumpsite are commonly sold in Enugu Urban market [44]. Elevated levels of Cd and Pb have been reported in vegetables cultivated in urban waste dumps of Kumasi, Ghana [16]. However, consumption of vegetables cultivated in heavy metals polluted soils has been reported to reduce life expectancy by 9 to 10 years [45]. Leafy vegetables exhibit preferential uptake of cadmium and lead from the soil [46]. The order of soil to plant transfer is Cd > Cu > Zn > Pb > As. Figure 5 also shows that heavy metal accumulation in dumpsite soil is more pronounced in the dry season. This can be expected since leaching which is the major mechanism of pollutant transport in the soil hardly occurs during the dry season. With the exception of copper and lead, all the other metals had high concentration at the core of the dumpsite in the dry season.

The leachability/mobility of heavy metals is usually inhibited by their affinity for certain substances in the soil such as clay, organic matter, hydrous oxides, etc. The pronounced variability in the spatial and lateral behaviours of the heavy metals can be attributed to the difference in their physicochemical characteristics. Heavy metal binding capacities is site specific and parameters relevant to binding at one site may be insignificant at another [13]. Another very crucial factor responsible for the high level of heavy metals accumulation in the waste dump is that the conditions prevailing in the dumpsite are not optimal for proper waste stabilization. The result is that organic fraction of disposed waste decomposes at a very slow rate.

### Geoaccumulation (I_geo_) and Pollution Indices (PI)

Geoaccumulation and pollution indices are used to assess the risks associated with heavy metals in the environment. From the pollution indices of the various heavy metals depicted in Figure 3, there is a moderate to extreme level of heavy metal pollution in the study area. Iron and manganese have PI values of 13.2 (dry season), 10.6 (rainy season) and 7.7 (dry season), 3.1 (rainy season) respectively. Apart from the rainy season PI value for manganese, the other PI values indicate extreme levels of soil pollution by these metals. Copper, arsenic, and nickel have PI values which indicate high level of pollution (2 ≤ PI ≤ 5). These heavy metals are most likely from sawdust and timber products disposed on the dumpsite [47]. The most commonly used wood preservatives are the copper chromium arsenic (CCA) salts. Though these metals have low mobility under normal conditions, under acidic conditions their mobility increases [48]. Salts of chromium, copper and arsenic can easily enter the human environment through improper disposal of sawdust. These metals are usually held tightly within the wood matrices but can get liberated during sawing. Moreover, sawdust has large surface areas which can facilitate desorption of these salts.

Zinc, cadmium and chromium have PI values between 1 and 2 which indicates moderate level of pollution. Only cobalt has a PI value less than 1 in both dry and rainy seasons. This implies that cobalt pollution in the dumpsite currently stands at a very low level. It can be seen from Figure 3 that the dry season PI values computed for most of the metals were much higher than rainy season PI values. This can be attributed to the fact that heavy metals tend to accumulate near the top soil during the dry season [49]. However, with the onset of rainy season, the metals are mobilized and dispersed by leachates. The pollution index gives the pollution status of the soil with respect to individual metals. Hence a composite value is needed to ascertain the overall status of the soil. The pollution loading index (PLI) meets that requirement. The PLI values for the soil are 1.706 for rainy season and 2.54 for dry season. PLI value greater than 1 signifies deterioration. It seems that conditions that favour heavy metals mobility prevail in the rainy season. A substantial degree of heavy metals attenuation via plant uptake occurs in the rainy season when plants and grasses are growing. As dry season approaches, grasses and remains of plant roots and shoots wither and are re-integrated into the soil giving rise to a cyclic sequence of up-take and deposition.

**Figure 6:**
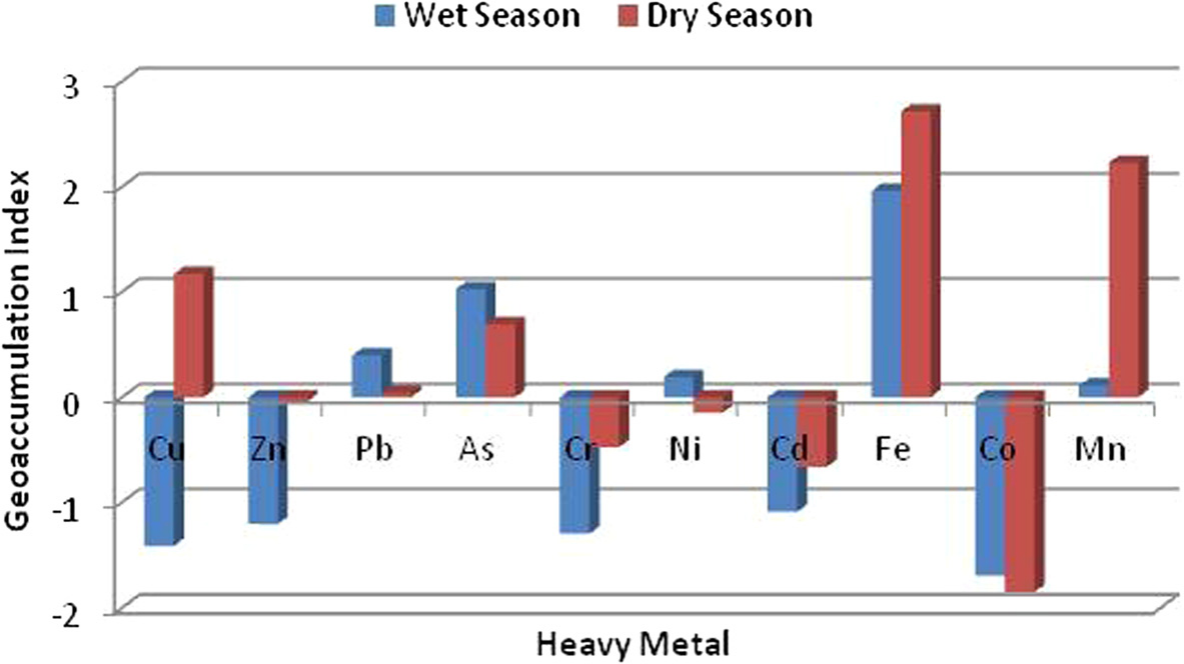
**Seasonal variation of geoaccumulation index.**

**Figure 7:**
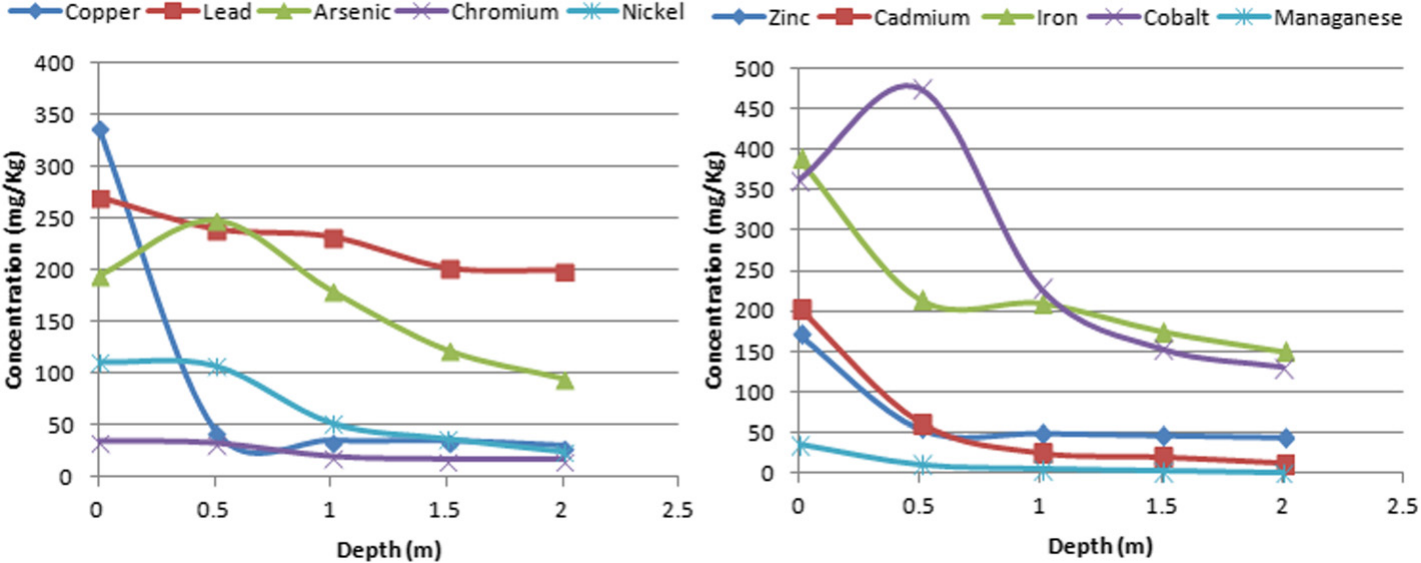
**Attenuation of heavy metals with soil depth.**

The values of I_geo_ obtained for the individual metals (Figure 6) serve to further buttress the results of the PI values. Iron, manganese, copper and arsenic have the highest I_geo_ values, while cobalt, chromium, cadmium and zinc have I_geo_ values less than zero. From the I_geo_ values, it can be deduced that the soil is currently uncontaminated with respect to cobalt, cadmium, chromium and zinc; moderately to heavily contaminated with respect to iron and manganese; and moderately contaminated with respect to copper, lead, arsenic and nickel. From Figure 7, it appears that a substantial concentration of heavy metals is confined within the first 1 m of soil depth. This suggests that the mobility of heavy metals is affected by the concentration in the top soil. The concentrations of most of the heavy metals, specifically, chromium, manganese, copper, nickel, cadmium and zinc approached a constant level at a depth of 1 m. On the other hand, the concentrations of arsenic, cobalt and iron continued to decrease even at a depth of 2 m. This phenomenon confirms the suggestion that individual heavy metals, move at different rates in the soil [50]. It also suggests that individual heavy metals move in plumes. Hence the plumes or copper, lead, chromium, nickel, manganese, cadmium, and zinc are lagging behind the plume of arsenic, iron and cobalt. The rate of movement of this plume is dependent on the loading of the heavy metals on the top soil and other site characteristics. It can further be deduced that the mobility of arsenic, cobalt and iron is most favoured by condition prevailing in this site. It is also likely that these heavy metals will breakthrough to groundwater faster than other metals.

Figure 7 also shows that copper is very reluctant to move and therefore has a tendency to accumulate in soil. The concentration of copper dropped from 330 mg/kg to less than 50 mg/kg in just 0.5 m of soil depth, and afterwards dropped no further. Zinc and cadmium also follow the same pattern to a smaller degree. Another reason for the high disparity between heavy metal concentration in the top soil and that in the soil below is that many of the heavy metals are still bound to waste materials in the waste dump. It is however, expected that as biological processes in the dumpsite progress, these heavy metals will be released from their parent sources into the soil [51]. When these metals are mobilized, they become more available for leaching and plant uptake, thereby increasing their environmental risk. This implies that waste dumpsites without impermeable linings as is common in developing countries should undergo remediation in order to reduce associated long term risks [52]. The high concentration of iron in the dumpsite as well as other locations investigated by various researchers is understandable. Iron is the second most abundant metal in the earth crust and has a wide range of domestic and industrial applications. Its relatively high activity makes it susceptible to corrosion. When iron corrodes, it forms a flaky and porous oxide so that particles from the parent material are easily detached and deposited in the environment. Iron is a very essential mineral and is a basic component of blood. However, excess intake of iron beyond the recommended dietary limits can cause health problems.

Other metals such as arsenic, cobalt, cadmium, chromium and nickel are extensively used in the industries either as raw materials, stabilizers, preservatives, catalysts or inhibitors. They form a major component of very common materials such as paints, pigments, ink, batteries, fungicides, pesticides, fertilizers, dyes, printer and photocopier toner, adhesives, paper etc. All these products and many more are disposed at dumpsites. Though arsenic is a component of most plants, its biological function has not been determined and the risk of arsenic transfer from plant to man is very low as plants cannot survive high doses of the metal. Table 4 shows that the ecological risk posed by heavy metals in the study location is high. About 91% of this risk is contributed by cadmium, whereas the risk posed by the other heavy metals is very low. The decreasing order of risk is: Cd > Co > Pb > Ni > Cu > Zn > Cr. There is therefore a need to discontinue waste disposal at this site and immediately embark on remediation measures in order to reduce environmental impact.

**Table 4:** **Ecological risk indices**

	**Copper**	**Zinc**	**Lead**	**Chromium**	**Nickel**	**Cadmium**	**Cobalt**	**Total**
**Rainy season**	0.90	0.14	2.31	0.15	1.37	*201.52*	15.19	221.58
**Dry season**	3.13	0.25	2.10	0.24	1.02	*215.53*	13.30	235.58
Level of contamination	Low	Low	Low	Low	Low	*High*	Low	High

Sources of cadmium at the dumpsite include cigarette butts, sewage, fertilizers, batteries, pigments, plastics and paints. The global adult tobacco survey estimated that 5.6% (4.7 million) of Nigerians smoke cigarette at an average daily consumption rate of 8 sticks [53]. This translates to 37.6 million cigarette stubs disposed at dumpsites on a daily basis or 14 billion cigarette butts annually. Other heavy metals contained in cigarette are lead and arsenic which are easily leached into the soil from the highly porous butts. It has been estimated that the lead, arsenic and cadmium contents of cigarette ranged from 0.02– 6.75 μg/g, 0.02– 0.71 μg/g [54] and 0.4 – 2.3 μg/g [55] respectively. However, only 10 to 20% of these heavy metals are inhaled by the smoker, while the rest is discharged into the environment [56]. Anthropogenic release of cadmium into the environment can also be attributed to the rise in use of nickel-cadmium rechargeable batteries in phones, torches, laptops, radio sets and other electronic gadgets. In Nigeria, most of these wastes are co-disposed with other low risk wastes in municipal waste dumps. Other metals associated with electronic wastes are: zinc used as screen coating; copper used as circuit board solder; lead used in cathode ray tubes, solders and batteries/accumulators; chromium used as metal coating; and nickel.

**Figure 8:**
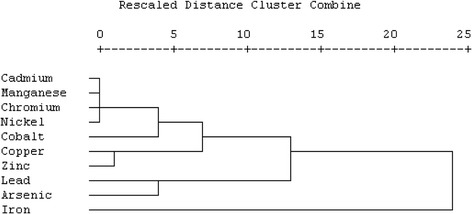
**Hierarchical clusters of heavy metals.**

An attempt at systematic source identification was made using hierarchical cluster analysis. It appears that cluster analysis was not very successful in delineating the heavy metals into sources of origin. This is partly because most waste materials contain a diversity of metals and non metals. Hence, these metals are found to co-exist with one another as a result of their varied and complementary industrial applications. However, a very careful examination of the clusters revealed groupings in terms of metal toxicity and essentiality to living things. Using hierarchical cluster analysis (Figure 8), the heavy metals can be grouped into four classes. Cluster 1 consists of cadmium, manganese, chromium, nickel and cobalt. All these apart from cadmium are essential minerals which, however, pose high risks when consumed in large doses. The major sources of these metals at the dumpsite are: paints, fertilizers, battery, photographic films, match, demolition waste, brake lining, etc. Cluster 2 consists of zinc and copper which are medium risk essential minerals possibly from batteries, printing ink, photocopier toner, fertilizers, cigarette butts, wood preservatives, copper wire, roofing sheets, cosmetics and pharmaceuticals. Cluster 3 consists of lead and arsenic which are confirmed carcinogens. The major sources of these metals are cigarette butts, paints, pigments, dyes, pharmaceuticals, battery and preservatives at the dumpsite. The only metal in cluster 4 is iron which can be classified as a low risk essential mineral.

## Conclusion

The status of soil in the dumpsite and environs has been heavily compromised due to indiscriminate disposal of untreated waste. Unfortunately, this dumpsite is the final resting place for all waste generated within the municipality. These heavy metals accumulate in plants and are subsequently transmitted to humans. They are also leached into groundwater by rainfall. In order to check groundwater contamination from this site, the dump should be converted to a constructed landfill with impermeable lining. This lining will serve as a barrier between leachate and groundwater.
